# miRNA–protein–metabolite interaction network reveals the regulatory network and players of pregnancy regulation in dairy cows

**DOI:** 10.3389/fcell.2024.1377172

**Published:** 2024-08-02

**Authors:** Bhaswati Chatterjee, Suman S. Thakur

**Affiliations:** ^1^ National Institute of Animal Biotechnology (NIAB), Hyderabad, India; ^2^ Centre for Cellular and Molecular Biology, Hyderabad, India

**Keywords:** dairy cow early pregnancy stage, miRNA–protein interaction, protein–metabolite interactions, protein–protein interaction, metabolite–metabolite interaction, miRNA–protein–metabolite interaction

## Abstract

Pregnancy is a complex process involving complex molecular interaction networks, such as between miRNA–protein, protein–protein, metabolite–metabolite, and protein–metabolite interactions. Advances in technology have led to the identification of many pregnancy-associated microRNA (miRNA), protein, and metabolite fingerprints in dairy cows. An array of miRNA, protein, and metabolite fingerprints produced during the early pregnancy of dairy cows were described. We have found the *in silico* interaction networks between miRNA–protein, protein–protein, metabolite–metabolite, and protein–metabolite. We have manually constructed miRNA–protein–metabolite interaction networks such as bta-miR-423-3p–IGFBP2–PGF2α interactomes. This interactome is obtained by manually combining the interaction network formed between bta-miR-423-3p–IGFBP2 and the interaction network between IGFBP2–PGF2α with IGFBP2 as a common interactor with bta-miR-423-3p and PGF2α with the provided sources of evidence. The interaction between bta-miR-423-3p and IGFBP2 has many sources of evidence including a high miRanda score of 169, minimum free energy (MFE) score of −25.14, binding probability (p) of 1, and energy of −25.5. The interaction between IGFBP2 and PGF2α occurs at high confidence scores (≥0.7 or 70%). Interestingly, PGF2α is also found to interact with different metabolites, such as PGF2α–PGD2, PGF2α–thromboxane B2, PGF2α–PGE2, and PGF2α–6-keto-PGF1α at high confidence scores (≥0.7 or 70%). Furthermore, the interactions between C3–PGE2, C3–PGD2, PGE2–PGD2, PGD2–thromboxane B2, PGE2–thromboxane B2, 6-keto-PGF1α–thromboxane B2, and PGE2–6-keto-PGF1α were also obtained at high confidence scores (≥0.7 or 70%). Therefore, we propose that miRNA–protein–metabolite interactomes involving miRNA, protein, and metabolite fingerprints of early pregnancy of dairy cows such as bta-miR-423-3p, IGFBP2, PGF2α, PGD2, C3, PGE2, 6-keto-PGF1 alpha, and thromboxane B2 may form the key regulatory networks and players of pregnancy regulation in dairy cows. This is the first study involving miRNA–protein–metabolite interactomes obtained in the early pregnancy stage of dairy cows.

## 1 Introduction

Pregnancy in dairy cows enables calf production used for breeding development and herd repair, along with milk maintenance for the dairy industry. The first 3 weeks after insemination comprise the most important phase of pregnancy as many lactating cows suffer from the loss of embryos before implantation, which escalates the economic burden on dairy farmers. Generally, early embryonic mortality may happen before the day 16 of gestation when corpus luteum life is not extended with the return to the estrus cycle (Szenci, 2021). During placentation, the pregnancy retention varied with progesterone and estradiol concentration, cow’s age, body condition, and service sire (Starbuck et al., 2004). The pregnancy retention decreased in cows with increased age and high body condition. Most of the pregnancies are maintained in cows with an average body condition (Starbuck et al., 2004). The lower rate of pregnancy retention has been detected in one service sire between 5 and 9 weeks. Interestingly, animals with two corpus lutea (CL) maintained fewer pregnancies; however, more data are required about the animals who had viable multiple embryos at the start of the detection of pregnancy (Starbuck et al., 2004). Multiple-service Holstein cows have reduced the success of embryo transfer if they had metritis in the early postpartum period (Estrada-Cortés et al., 2019).

Bovine pregnancy is conventionally detected by rectal palpation (between 40 and 60 days after artificial insemination), ultrasonography (between 25 and 30 days after artificial insemination), or by changes in progesterone concentration in blood (serum) or milk (between 18 and 24 days after artificial insemination) (Szenci, 2021), suggesting their ability of accurate detection after 3 weeks of pregnancy. However, excess contact may increase the chances of fetus or embryo loss (Franco et al., 1987; Thurmond and Picanso, 1993; Thompson et al., 1994). Transrectal ultrasound scanning is the gold standard for the detection of pregnancy; however, this involves expertise and use of expensive equipment and can be performed after 28 days post-artificial insemination (AI) (Johnston et al., 2018). Furthermore, estrus visualization with the help of tail paint/heat pads is labor-intensive and not dependable due to silent and/or missed heats (Johnston et al., 2018).

The placental lactogen, pregnancy-specific protein B, bovine pregnancy-associated glycoprotein, and concentration of progesterone in milk were used for the detection of pregnancy in cows. However, these tests gave high false positives/high false negatives, and they differed in an individual’s serum concentration and were also present in different animal diseases (Pyo et al., 2003). Many diagnostic methods/tests for cow pregnancy detection were made, including pregnancy-associated glycoprotein (PAG) ELISA (Green et al., 2005; Barbato et al., 2017; Barbato et al., 2022), pregnancy-specific protein B (PSPB) radioimmunoassays (Humblot et al., 1988; Romano and Larson, 2010), early conception factor (ECF) lateral-flow assay (Cordoba et al., 2001; Ambrose et al., 2007), immunoassays regarding progesterone (Nebel et al., 1987), and in-line progesterone sensor (Friggens et al., 2008)-based pregnancy tests. However, these tests had several shortcomings and were not popular for detecting the early stages of pregnancy.

The absence of a reliable method for the detection of early pregnancy in cows decreases overall productivity, increases the calving interval, and causes a high economic burden to the dairy industry. The estrous cycle of bovine is approximately 21 days; therefore, efforts are being made for the identification of pregnancy biomarkers that detect the pregnancy before 21 days post-artificial insemination in a less stressful and less invasive way, thereby providing an opportunity to rebreed in the following estrus cycle. Generally, early embryonic death results 16 days post-insemination (Johnston et al., 2018); therefore, an early and accurate pregnancy diagnosis is most important.

The early diagnosis of cattle pregnancy is important, leading to the surveillance of the breeding outcome and shortening the calving interval. The state of pregnancy is accompanied by changes in the expression of miRNAs, proteins, metabolites and their abundances. The establishment of dairy cow’s genomic, proteomic, and metabolomic databases has led to the successful identification of suitable miRNAs, proteins, and metabolite fingerprints of their pregnancy. The high stability of microRNAs (miRNAs) renders them potential non-invasive biomarkers of diseases (Mitchell et al., 2008; Williams et al., 2013; Casey et al., 2015) with their association with different diseases, such as cancer, heart diseases, and diseases involving the autoimmune system, as well as in pregnancy-related contexts (Miura et al., 2010; Wu et al., 2011; Haider et al., 2014; He et al., 2015).

The “omics” technologies are capable of analyzing different aspects of the organism at genomic, transcriptomic, proteomic, and metabolomic levels (McGettigan et al., 2016). The synergies between these high-throughput technologies hold the key to maximizing the efficiency of the early detection of pregnancy. The last decade has shown significant updates in the field of proteomics, leading to an increased understanding of biological pathways affected by different diseases and physiological conditions (Yates, 2019). Interestingly, the growth of proteomic and metabolomic technologies in animal biology has enabled the global analysis of the proteome and metabolome of biological/clinical samples, including the detection of potential biomarkers that would be useful for the early detection of disease and the welfare, safety, and quality of animal products (Talamo et al., 2003; Bendixen et al., 2011; Turk et al., 2012; Ceciliani et al., 2014).

There are pregnancy-associated microRNAs (miRNAs), proteins, and metabolites that are differentially regulated during the early stages of pregnancy in dairy cows. This review incorporates important early pregnancy-associated miRNAs, proteins, and metabolites based on the literature. We aim to find miRNA–protein–metabolite interactomes formed during the early stages of pregnancy in dairy cows by manually integrating the miRNA–protein interaction network and protein–metabolite interaction networks formed from miRNAs, proteins, and metabolites associated with the early stages of pregnancy in dairy cows.

## 2 Early stage pregnancy-associated fingerprints of dairy cows

### 2.1 MicroRNA fingerprints associated with early pregnancy stages in dairy cows

MicroRNAs (miRNAs) can govern the expression of genes post-transcriptionally. They are also involved in pregnancy regulation in humans and animals (Miura et al., 2010; Morales Prieto and Markert, 2011; Li et al., 2012; Fu et al., 2013; Morales-Prieto et al., 2013; Ioannidis and Donadeu, 2016; Cai et al., 2017). To find the role of miRNAs as the biomarkers for early pregnancy diagnosis, Hong and co-workers profiled the circulating miRNAs in normal and 30 days of pregnancy and found differentially expressed miRNAs between them, including bta-miR-146b, bta-miR-193b, bta-miR-27b, bta-miR-197, bta-miR-26b, bta-miR-450b, bta-miR-339a, bta-miR-326, bta-let-7a-3p, bta-miR-484, bta-miR-486, bta-miR-423-3p, and bta-miR-92a (Markkandan et al., 2018). Furthermore, the upregulation of plasma miR-26a during early pregnancy in heifers suggested this as an early pregnancy biomarker (Ioannidis and Donadeu, 2016). Furthermore, using small RNA sequencing and RT-qPCR profiling, miRNAs such as let-7f, let-7c, miR-30c, miR-101, miR-26a, miR-205, and miR-143 increased in day 60 pregnant cows compared to non-pregnant cows. Interestingly, the level of miR-26a was found to be increased on day 8 of pregnant cows, suggesting their role in early pregnancy (Ioannidis and Donadeu, 2017).

The serum of pregnant cows contained differentially expressed miRNAs including miR-433, miR-487b, miR-495-3p, miR-376b-3p, and miR-323a-3p which were homologous to human pregnancy-associated C14MC miRNAs, suggesting their potential roles in early pregnancy (Gebremedhn et al., 2018). Another study has found an increase in bta-miR-221 and bta-miR-320a in 8, 12, and 16 weeks of pregnancy in dairy cows (Lim et al., 2021). The miRNA fingerprints of the early stage of pregnancy in dairy cows are summarized in Table 1.

**TABLE 1:** Summary of early pregnancy-associated important microRNAs of dairy cows.

S. no	Pregnancy-associated microRNA (miRNA)	Sample	(Days/months) of pregnancy	Level	Mode of measurement/validation	Study design			Enrolled population	Reference
						Retrospective	Prospective	Cross-sectional		
**1**	miR-26a	Plasma	Days 16–24 of pregnancy	Upregulated	Illumina small-RNA sequencing and RT-qPCR	• Cycling Holstein-Friesian heifers were estrus-synchronized using progesterone; buserelin on CIDR insertion; and cloprostenol after CIDR insertion	• After 48 h of estrus-synchronized insemination or sham-insemination of animals and again 24 h later • Blood collection was on days 0, 8, and 16 from all animals. Additional samples were collected on day 24 from the pregnant group • Exclusion from the study of five animals that did not become pregnant • Pregnancy was confirmed twice on days 35 and 60 by trans-rectal ultrasound • Validation of miRNA: 16 cycling, 14–17-month-old Holstein-Friesian heifers were estrus-synchronized and inseminated. Blood collection was on days 0, 16, and 24 and processed for RT-qPCR analysis		Number of controls (non-pregnant group): 8 Number of cases (pregnant group): 16	Ioannidis and Donadeu (2016)
**2**	miR-433 and miR-487b	Serum	Days 19 and 24 of pregnancy, respectively	Upregulated	Custom PCR arrays and cDNA synthesis	• Lactating Holstein-Friesian cows, at 50–80 days *postpartum* were kept under the same feeding and farm conditions with the free-stall housing system for at least 6 consecutive months • Cows with a blind quarter were excluded	• Cows in the first breeding of the lactation during experiments were used • Cows were estrous-synchronized and inseminated with frozen semen • Blood samples were collected 19 and 24 days post-insemination • Status of pregnancy determined 35 days post-insemination via ultrasonography		Total: 154 lactating Holstein-Friesian cows	Gebremedhn et al. (2018)
**3**	miR-495-3p, miR-376b-3p, and miR-323a-3p	Serum	Day 24 of pregnancy	Downregulated	Custom PCR arrays and cDNA synthesis	• Lactating Holstein-Friesian cows, at 50–80 days *postpartum*, were kept under the same feeding and farm conditions with the free-stall housing system for at least 6 consecutive months • Cows with a blind quarter were excluded	• Cows in the first breeding of the lactation during experiments were used • Cows were estrous-synchronized and inseminated with frozen semen • Blood samples were collected 19 and 24 days post-insemination. • Status of pregnancy determined 35 days post-insemination via ultrasonography		Total: 154 lactating Holstein-Friesian cows	Gebremedhn et al. (2018)
**4**	bta-miR-221 and bta-miR-320a	Plasma	8, 12, and 16 weeks of pregnancy	Increased	Small-RNA sequencing kit		• A total of 30 dairy cows were used • Non-pregnant cows were excluded from the analysis • Blood samples were collected from animals (n = 12) on 0, 4, 8, 12, and 16 weeks of pregnancy • Pregnancy was detected by palpation *per rectum* between days 50 and 60 after artificial insemination or ultrasonic examination		Total of 30 dairy cows were used	Lim et al. (2021)
**5**	let-7f, let-7c, miR-30c, miR-101, miR-26a, miR-205, and miR-143	plasma	Day 60 of pregnancy Furthermore, miR-26a identified on day 8	Increased	Small-RNA sequencing and RT-qPCR		• Eleven Holstein-Friesian heifers were estrus-synchronized and artificially inseminated • Pregnancy confirmed via trans-rectal ultrasound on days 35 and 60 post-insemination • Plasma samples were collected on days 0, 8, 16, and 60 post-insemination		Eleven Holstein-Friesian heifers, 14–17 months old, were used for the study	Ioannidis and Donadeu (2017)
**6**	bta-miR-146b, bta-miR-27b, bta-miR-26b, bta-miR-450b, and bta-let-7a-3p	Whole blood	30 days of pregnancy group	Upregulated	Small-RNA sequencing kit			Whole blood samples of normal and 30 days of pregnancy from Holstein cow were collected	Three healthy dairy cows of normal and 30 days of pregnancy were taken	Markkandan et al. (2018)
**7**	bta-miR-193b, bta-miR-197, bta-miR-339a, bta-miR-326, bta-miR-484, bta-miR-486, bta-miR-423-3p, and bta-miR-92a	Whole blood	30 days of pregnancy group	Downregulated	Small-RNA sequencing kit			• Whole blood samples of normal and 30 days of pregnancy from Holstein cow were collected	Three healthy dairy cows of normal and 30 days of pregnancy were taken	Markkandan et al. (2018)

### 2.2 Protein fingerprints associated with early pregnancy stages in dairy cows

Proteins such as methylmalonyl-CoA mutase, hemoglobin subunit beta, T-complex protein 1 subunit theta, apolipoprotein A-II, apolipoprotein AI, albumin, putative helicase MOV-10, aspartate aminotransferase, vacuolar protein-sorting-associated protein 36, Tuftelin-interacting protein 11, transcription factor IIF subunit 2, translation initiation factor eIF-2B subunit beta, and annexin A9 were found in pregnant cows. Annexin A9 was related to the early development of the embryo. In addition, LDH was also found in early pregnant cows (Mojsym et al., 2022). Interestingly, alpha-1 G and lactoferrin/lactotransferrin were increased in pregnant cow milk 35 days after insemination, were expressed in a pregnancy-associated manner, and probably were biomarkers of early pregnancy (Han et al., 2012). Furthermore, bovine pregnancy-associated protein (bPAP) is also found to be related to pregnancy, as found in pregnant Holstein cows (Pyo et al., 2003).

A pilot study comparing pregnant and non-pregnant heifers during the peri-implantation period showed that the levels of expression of proteins such as growth arrest-specific protein 1 (GAS1), beta-2-glycoprotein 1 (APOH), follistatin-related protein 1 (FSTL1), and fibulin-1 were increased, while the levels of serotransferrin (TF), F1MLW8, and immunoglobulin light chain (IGL@) were decreased, and these may be used for the detection of early pregnancy (Ruiz Álvarez et al., 2023).

Studies were carried out using two-dimensional-fluorescence difference gel electrophoresis (2D DIGE) and MALDI-TOF mass spectrometry for the serum of pregnant and non-pregnant cattle, and it was found that proteins such as the conglutinin precursor, modified bovine fibrinogen, and IgG1 were upregulated, while complement component 3, bovine fibrinogen, and IgG2a were downregulated in the pregnant cattle serum (Lee et al., 2015). Interestingly, interferon-stimulated gene-15 ubiquitin-like modifier (ISG15) protein, myxovirus resistance (MX1 and MX2) proteins, and oligoadenylate synthetase-1 (OAS1) on blood neutrophils were found to be of higher abundance on day 18 after AI, and these were also supported by gene expression studies. This indicates that these proteins are important for the establishment of pregnancy and may be the biomarker for the diagnosis of cow pregnancy (Panda et al., 2020).

Studies have shown that APOB, SPADH1, PLIN2, LPO, PIGR, PGD, QSOX1, MUC1, SRPRA, MD2, GAPDH, FOLR1, GPRC5B, and HHIPL2 were differentially expressed between the proteomes of pregnant (day 21) milk whey and estrous cycle (day 21) milk whey. These proteins were also the potential biomarkers of early pregnancy (Johnston et al., 2018).

Rawat et al (2016) found that during early pregnancy (16–22 days), differentially expressed proteins such as Mannan-binding protein (MBP), haptoglobin, SERPINB3-like, uromodulin, cathelicidin, uteroglobin, vitamin-binding protein, and insulin-like growth factor-binding protein II (IGFBP-II) were increased in Karan Fries (KF) heifers. The protein fingerprints of the early stage of pregnancy in dairy cows are summarized in Table 2.

**TABLE 2:** Summary of important protein fingerprints of early stages of pregnancy in dairy cows.

S. No	Pregnancy-associated protein	Sample	Pregnancy (day/month)	Level	Mode of measurement	Study design			Enrolled population	Reference
						Retrospective	Prospective	Cross-sectional		
1	Methylmalonyl-CoA mutase, hemoglobin subunit beta, T-complex protein 1 subunit theta, apolipoprotein A-II, apolipoprotein AI, albumin, putative helicase MOV-10, aspartate aminotransferase, vacuolar protein-sorting-associated protein 36, tuftelin-interacting protein 11, transcription factor IIF subunit 2, translation initiation factor eIF-2B subunit beta, annexin A9, and LDH	Saliva and plasma	3–4 month-pregnant cows	-	2D electrophoresis and Ultraflex III MALDI TOF/TOF spectrometer	• Selected Holstein-Friesian cows were at least 60 days after the last parturition		• Progress of pregnancy was evaluated by the date of artificial insemination and per rectal USG • Pregnancy established for 3–4 months • Control (non-pregnant) animals were at a similar age as pregnant individuals (4–8 years old) • Both saliva and plasma from control and pregnant groups were collected on the same day in the morning before feeding	Number of control (non-pregnant) groups: 4 Number of cases (pregnant group):8	Mojsym et al. (2022)
2	Alpha-1 G and lactotransferrin	milk	Pregnant Holstein dairy cattle 35 days after artificial insemination (AI)	Increased	2-DE and MALDI-TOF MS		• Milk samples were obtained from five pregnant Holstein dairy cattle 35 days after artificial insemination (AI) and from five non-pregnant cattle • For confirming proteomics results with Western blot analysis, milk samples were collected from another pregnant cattle		Number of control (non-pregnant) groups: 5 Number of cases (pregnant group): 5	Han et al. (2012)
3	Growth arrest-specific protein 1 (GAS1), beta-2-glycoprotein 1 (APOH), follistatin-related protein 1 (FSTL1), and fibulin-1	Serum		Increased	2-DE/iTRAQ–MALDI–TOF-TOF	• Cows (n = 40) were Aberdeen Angus heifers, synchronized by inserting progesterone-releasing insert (CIDR) prior to artificial insemination (AI) • D + Cloprostenol was injected upon CIDR removal and GnRH boosts were applied at 10 days and 1 day before AI.		• AI was performed using commercial semen • Serum samples were classified as pregnant (P) or non-pregnant (NP)	Cows (n = 40) Number of control (non-pregnant) groups: 21 Number of cases (pregnant group):19	Ruiz Álvarez et al. (2023)
4	Serotransferrin (TF), A5PK72, F1MLW8, and immunoglobulin light chain (IGL@)	Serum		Decreased	2-DE/iTRAQ–MALDI–TOF-TOF	• Cows (n = 40) were Aberdeen Angus heifers, synchronized by inserting progesterone-releasing insert (CIDR) prior to artificial insemination (AI) • D + Cloprostenol was injected upon CIDR removal and GnRH boosts were applied at 10 days and 1 day before AI.		• AI was performed using commercial semen • Serum samples were classified as pregnant (P) or non-pregnant (NP)	Cows (n = 40) Number of control (non-pregnant) groups: 21 Number of cases (pregnant group):19	Ruiz Álvarez et al. (2023)
5	Conglutinin precursor, modified bovine fibrinogen, and IgG1	Serum	Pregnant Holstein cattle at day 21 after AI	Upregulated	2D DIGE and MALDI-TOF			• Serums of two pregnant Holstein cattle at day 21 after AI and those of two non-pregnant cattle for analyzing of proteomics	Number of control (non-pregnant) groups: 2 Number of cases (pregnant group): 2	Lee et al. (2015)
6	Hemoglobin, complement component 3, bovine fibrinogen, and IgG2a	Serum	Pregnant Holstein cattle at day 21 after AI	Downregulated	2D DIGE and MALDI-TOF			• Serums of two pregnant Holstein cattle at day 21 after AI and those of two non-pregnant cattle for analyzing of proteomics	Number of control (non-pregnant) groups: 2 Number of cases (pregnant group): 2	Lee et al. (2015)
7	Mannan-binding protein (MBP), haptoglobin, SERPINB3-like, uromodulin, cathelicidin, uteroglobin, vitamin-binding protein, and insulin-like growth factor-binding protein II (IGFBP-II)	Urine	Days 0, 16, 22, and 35 upto day 60 of pregnancy in Karan Fries (KF) heifers	Increased	2D DIGE and LC–MS/MS		• Urine was collected from individual Karan Fries heifers (n = 6) on different days of pregnancy (0, 16, 22, and 35 days) • Day 0 represents the control [urine collection before artificial insemination (AI)] • Following AI, urine was collected until day 60 of pregnancy		Total: 6	Rawat et al. (2016)
8	Interferon-stimulated gene-15 ubiquitin-like modifier (ISG15) protein, myxovirus resistance (MX1 and MX2) proteins, and oligoadenylate synthetase-1 (OAS1)	Blood neutrophils	10th, 18th, and 36th days post-AI	High	LC–MS/MS	• Karan Fries cows were offered *ad lib* green fodder, water, and calculated amount of the concentrate mixture	• Blood samples were collected on four different days, i.e., days 0th, 10th, 18th, and 36th post-AI for each cow • At day 45 after AI, pregnancy diagnoses were performed • Out of 20 cows, 9 were confirmed as pregnant, and these pregnant samples were used for further study. Day 0 was considered as non-pregnant		Total: 20	Panda et al. (2020)
9	APOB, SPADH1, PLIN2, PIGR, PGD, QSOX1, MUC1, SRPRA, and MD2	Milk whey	21 days post-AI	Increased	LC–MS/MS	• Estrous cycles of 81 multiparous Holstein-Friesian dairy cows were synchronized • Intra-vaginal progesterone-releasing device (CIDR) was inserted in the vagina of each cow • Each cow simultaneously received gonadotropin-releasing hormone. Seven days later, the cows received injection of prostaglandin and either heat patches or tail paint were applied on the tail head of the cows, as aids to detect estrus		• All cows went through one (control) estrous cycle • On day 21 of the control cycle (i.e., day 0 of the following cycle), milk samples for proteomic analyses were collected • Seventy-four cows were artificially inseminated 12 h, following observation of estrus (day 0) • Milk samples for proteomic analyses were collected 21 days post-AI (day 21) • Forty-five cows were confirmed pregnant via ultrasound scanning on day 35 post AI (day 35) • Ten of these cows were selected for use in the present study	• Total: 81 • Seventy-four cows were artificially inseminated • Forty-five cows were confirmed pregnant • Ten of these cows were selected for study	Johnston et al. (2018)
10	LPO, GAPDH, FOLR1, GPRC5B, and HHIPL2	Milk whey	21 days post- AI	Decreased	LC–MS/MS	• Estrous cycles of 81 multiparous Holstein-Friesian dairy cows were synchronized • Intra-vaginal progesterone-releasing device (CIDR) was inserted in the vagina of each cow • Each cow simultaneously received gonadotropin-releasing hormone. Seven days later, the cows received injection of prostaglandin and either heat patches or tail paint were applied on the tail head of the cows, as aids to detect estrus		• All cows went through one (control) estrous cycle • On day 21 of the control cycle (i.e., day 0 of the following cycle), milk samples for proteomic analyses were collected • Seventy-four cows were artificially inseminated 12 h following observation of estrus (day 0) • Milk samples for proteomic analyses were collected 21 days post-AI (day 21) • Forty-five cows were confirmed pregnant via ultrasound scanning on day 35 post-AI (day 35) • Ten of these cows were selected for use in the present study		Johnston et al. (2018)

### 2.3 Metabolite fingerprints associated with early pregnancy stages in dairy cows

Understanding the metabolic global changes in pregnant dairy cows was undertaken by metabolomics studies during early pregnancy, that is, on days 0, 17, and 45 after artificial insemination (AI). It was found that metabolic profiles on days 17 and 45 were significantly different from day 0. In addition, there were no significant differences in metabolic profiling on days 17 and 45. The alpha-linolenic acid (ALA) level was low on days 17 and 45 of pregnancy. Furthermore, low levels of some important metabolites such as L-dopa, L-tyrosine, tetrahydrobiopterin, 2,5-diaminopyrimidine nucleoside triphosphate, folic acid, pantothenic acid, and inositol 1, 3, 4-trisphosphate (IP3), and metabolites involved in thiamine metabolism, TCA cycles, folate biosynthesis pathway, one-carbon metabolism, cysteine and methionine metabolism, purine metabolism, and pentose and glucuronate interconversion pathways were observed on day 17 and/or day 45 of pregnancy (Guo and Tao, 2018).

Interestingly, at days 15 and 18 of gestation, the prostaglandin (6-keto PGF_1α_, PGF_2α_, PGE_2_, PGD_2_, and TXB_2_) levels increased more than those found on day 12 of gestation, which is important for early embryonic development. The increase was in the order of 6-keto PGF_1α_ ⟩ PGF_2α_ ⟩ PGE_2_ ⟩ PGD_2_⟩ and TXB_2_. The concentration of 6-keto PGF_1α_ was found to be highest on day 15 of gestation (Ulbrich et al., 2009). The metabolite fingerprints of the early stages of pregnancy in dairy cows are summarized in Table 3.

**TABLE 3:** Summary of important metabolite fingerprints of the early stage of pregnancy in dairy cows.

S. No.	Pregnancy-associated metabolite	Sample	Level of metabolite	Pregnancy	Mode of measurement	Statistical method	Study design			Enrolled population	References
							Retrospective	Prospective	Cross-sectional		
1	Alpha-linolenic acid, L-dopa, L-tyrosine, tetrahydrobiopterin, 2,5-Diaminopyrimidine nucleoside triphosphate, folic acid, pantothenic acid, and inositol 1, 3,4-trisphosphate (IP3)	Plasma	Decreased	Days 17 and 45 after artificial insemination	HPLC–QTOF/MS	Multivariate statistical analysis, PCA, OPLS-DA, Mann–Whitney U test, and Benjamini and Hochberg procedure		• Holstein cows were in their second lactation • Estrus-synchronized • Divided into three groups (Group A, Group B, and Group 3) • Each group of cows was artificial insemination on the same day • Group A: 12 plasma samples from dairy cows were collected on day 0 at the time of AI. • Group B: On day 17, 11 plasma samples from dairy cows were collected and confirmed to be from corresponding pregnant cows on day 45 • Group C: Fourteen plasma samples were collected from pregnant dairy cows on day 45		Number of controls: 12 Number of cases: 25	Guo and Tao (2018)
2	PGE2, PGD2, TXB2, PGF2α, and 6-keto PGF1α	Uterus fluid	Increased	Days 15 and 18 of gestation	LC–MS/MS	Least-square regression		Cyclic simmental heifers of 23 months of age were cycle-synchronized • Blood samples taken on synchronization day, day 0 of the estrous cycle, and just before slaughtering • Pregnant groups of animals were artificially inseminated with sperm and slaughtered at days 12, 15, and 18 of gestation, respectively, (n = 5 per group) • For these three groups, control groups (n = 5–7 per group) were inseminated with the supernatant of centrifuged sperm from the same bull and slaughtered at days 12, 15, and 18 of the estrous cycle, respectively • If no intact conceptus was detected in case of AI, then those animals were excluded		Number of controls: (n = 5–7 per group), 3 groups Number of cases: (n = 5 per group), 3 groups	Ulbrich et al. (2009)

## 3 Results

### 3.1 Gene Ontology analysis and pathway enrichment analysis of circulating miRNAs of the early pregnancy stages of dairy cows

To know about the target genes of differentially expressed miRNA fingerprints listed in Table 1, we used miRNet, a web-based platform tool (http://www.mirnet.ca/) (Fan et al., 2016; Fan and Xia, 2018). The miRNet (Fan et al., 2016) incorporates well-annotated databases such as miRTarBase (Hsu et al., 2011), TarBase (Vergoulis et al., 2012), miRecords (Xiao et al., 2009), SM2miR (Liu et al., 2013), Pharmaco-miR (Rukov et al., 2014), miRanda (Betel et al., 2010), miR2Disease (Jiang et al., 2009), PhenomiR (Ruepp et al., 2012), StarBase (Yang et al., 2011), EpimiR (Dai et al., 2014), and miRDB (Wong and Wang, 2015). Supplementary Table S1 summarizes the target genes of differentially expressed miRNA fingerprints listed in Table 1 using miRNet (http://www.mirnet.ca/) (Fan et al., 2016; Fan and Xia, 2018), with a degree filter cutoff of default value 1 using the well-annotated miRanda database (Betel et al., 2010) and MFE scores that explain the binding affinity between miRNAs and their target genes (Rath et al., 2016).

Furthermore, the resulting target genes (Supplementary Table S1) of the miRNA fingerprints listed in Table 1 were subjected to Gene Ontology biological process (GO-BP) (Supplementary Table S2), Gene Ontology molecular function (GO-MF) (Supplementary Table S3), and Gene Ontology cellular component (GO-CC) (Supplementary Table S4) analyses using the PANTHER tool (Mi and Thomas, 2009; Mi et al., 2019; Thomas et al., 2022) and KEGG pathway (Supplementary Table S5) analyses using the DAVID database/tool (Dennis et al., 2003; Huang et al., 2007; Sherman et al., 2007; Sherman et al., 2022), with the false discovery rate (FDR) of 0.05 as a significance threshold. Therefore, the resulting target genes of the miRNA fingerprints were enriched in biological processes such as the regulation of cAMP-mediated signaling, histone deacetylation, positive regulation of neurogenesis, positive regulation of cell differentiation, positive regulation of protein kinase activity, regulation of cell development, positive regulation of developmental process, regulation of cell differentiation, and regulation of developmental process (Supplementary Table S2). Among the identified molecular functions, growth factor binding, and water transmembrane transporter activity were enriched (Supplementary Table S3). Furthermore, the enriched cellular components were composed of trans-Golgi network membrane, Golgi apparatus, cytoplasmic vesicle, vesicles, and cytoplasm (Supplementary Table S4).

In addition, the resulting target genes of the miRNA fingerprints were enriched in pathways such as vasopressin-regulated water reabsorption, Ras signaling pathway, focal adhesion, T-cell receptor signaling pathway, TNF signaling pathway, Wnt signaling pathway, Rap1 signaling pathway, MAPK signaling pathway, and calcium signaling pathway exported from KEGG pathway analysis (Supplementary Table S5).

### 3.2 Protein–protein, protein–metabolite, and metabolite–metabolite interactions between protein and metabolite fingerprints of early pregnancy stages in dairy cows

**FIGURE 1:**
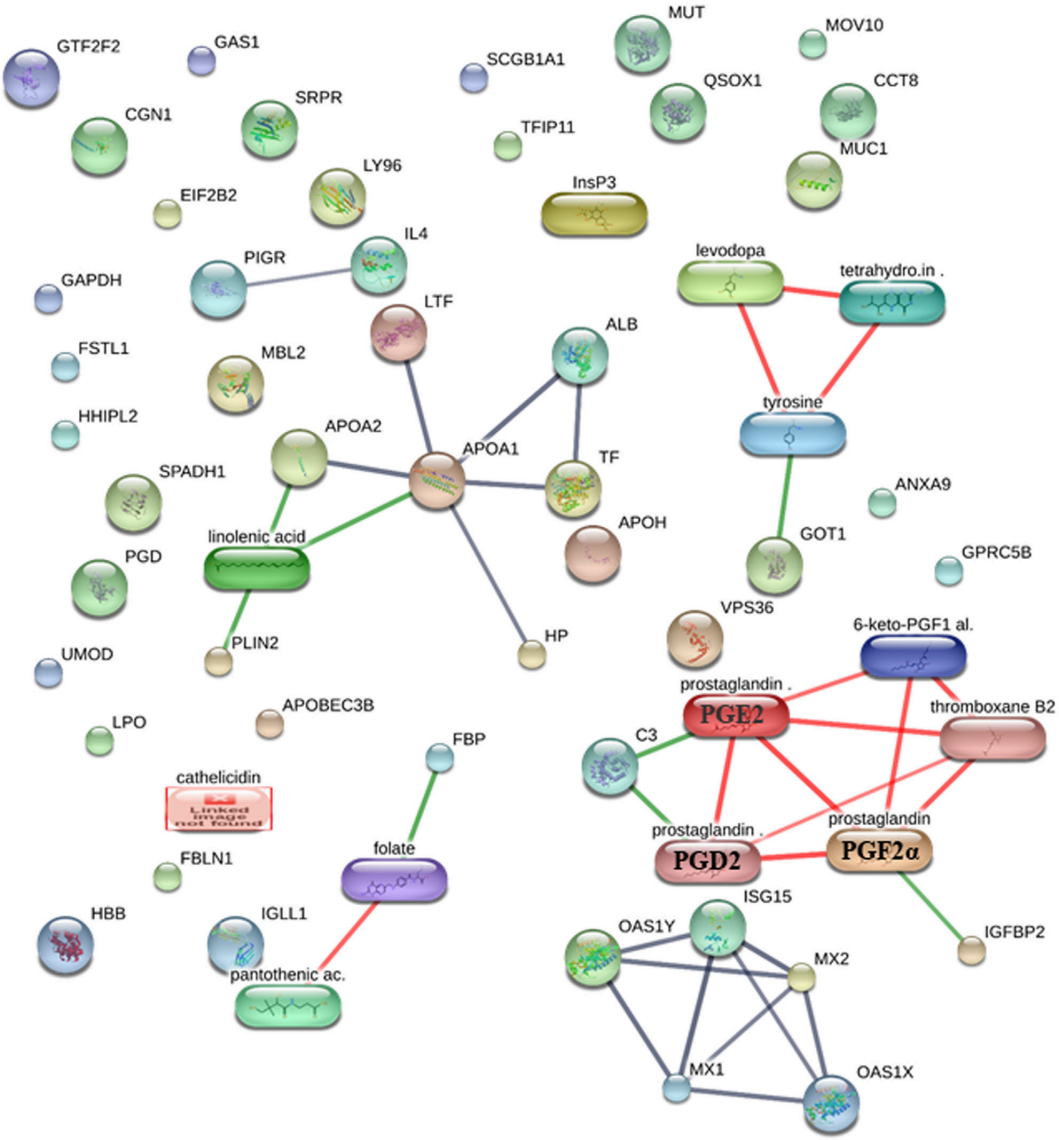
Protein–protein, protein–metabolite, and metabolite–metabolite interactions among protein and metabolite fingerprints of the early stage of pregnancy in dairy cows at high confidence scores. The protein–protein interactions are represented in gray, protein–metabolite interactions are represented in green, and metabolite–metabolite interactions are represented in red.

Using the STITCH database (Szklarczyk et al., 2016), we were able to find and identify the protein–protein, protein–metabolite, and metabolite–metabolite interactions between protein and metabolite fingerprints of the early stage of pregnancy in dairy cows at high confidence scores (≥0.7 or 70%) (Figure 1; Supplementary Table S6). The STITCH database incorporates the details from text mining, co-occurrence, co-expression, experiments, gene fusion, neighborhood, predictions, and databases (Szklarczyk et al., 2016).

We saw that the protein fingerprints form protein–protein interactions with high confidence, such as MX1–ISG15 (a high confidence score of 0.992 or 99.2%, including the scores from experiments, text mining, and co-expression), ISG15–MX2 (a high confidence score of 0.958 or 95.8%, including the scores from experiments, text mining, and co-expression), ALB–APOA1 (a high confidence score of 0.949 or 94.9%, including the scores from databases, text mining, and co-expression), APOA2–APOA1 (a high confidence score of 0.934 or 93.4%, including the scores from databases and co-expression), MX1–OAS1Y (a high confidence score of 0.929 or 92.9%, including the scores from experiments, text mining, and co-expression), TF–APOA1 (a high confidence score of 0.92 or 92%, including the scores from databases, text mining, and co-expression), APOA1–LTF (a high confidence score of 0.92 or 92%, including the scores from databases, text mining, and co-expression), OAS1Y–MX2 (a high confidence score of 0.911 or 91.1%, including the scores from experiments, text mining, and co-expression), OAS1X–MX2 (a high confidence score of 0.91 or 91%, including the scores from experiments, text mining, and co-expression), ISG15–OAS1Y (a high confidence score of 0.907 or 90.7%, including the scores from text mining and co-expression), OAS1X–MX1 (a high confidence score of 0.895 or 89.5%, including the scores from experiments, text mining, and co-expression), ALB–TF (a high confidence score of 0.892 or 89.2%, including the scores from text mining and co-expression), HP–APOA1 (a high confidence score of 0.813 or 81.3%, including the scores from experiments, text mining, and co-expression), OAS1X–ISG15 (a high confidence score of 0.804 or 80.4%, including the scores from text mining and co-expression), MX1–MX2 (a high confidence score of 0.804 or 80.4%, including the scores from homology, text mining, and co-expression), and PIGR–IL4 (a high confidence score of 0.70 or 70%, including the scores from text mining) (Figure 1; Supplementary Table S6).

The metabolites’ fingerprints form metabolite–metabolite interactions at high confidence such as prostaglandin (prostaglandin E2 or PGE2)–prostaglandin (PGF2α) (a high confidence score of 0.998 or 99.8%, including the scores from experiments, databases, homology, and text mining), prostaglandin (PGF2α)–prostaglandin (prostaglandin D2 or PGD2) (a high confidence score of 0.99 or 99%, including the scores from databases, homology, and text mining), levodopa–tetrahydrobiopterin (a high confidence score of 0.975 or 97.5%, including the scores from databases and text mining), prostaglandin (prostaglandin E2 or PGE2)–prostaglandin (prostaglandin D2 or PGD2) (a high confidence score of 0.97 or 97%, including the scores from databases, homology, and text mining), 6-keto-PGF1α–thromboxane B2 (a high confidence score of 0.961 or 96.1%, including the scores from text mining), levodopa–tyrosine (a high confidence score of 0.96 or 96%, including the scores from databases, homology, and text mining), prostaglandin (prostaglandin E2 or PGE2)–thromboxane B2 (a high confidence score of 0.938 or 93.8%, including the scores from text mining), tetrahydrobiopterin–tyrosine (a high confidence score of 0.933 or 93.3%, including the scores from databases and text mining), prostaglandin (PGF2α)–6-keto-PGF1α (a high confidence score of 0.923 or 92.3%, including the scores from homology and text mining), prostaglandin (PGF2α)–thromboxane B2 (a high confidence score of 0.92 or 92%, including the scores from text mining), pantothenic acid–folate (a high confidence score of 0.857 or 85.7%, including the scores from experiments and text mining), prostaglandin (prostaglandin E2 or PGE2)–6-keto-PGF1α (a high confidence score of 0.804 or 80.4%, including the scores from homology and text mining), and prostaglandin (prostaglandin D2 or PGD2)–thromboxane B2 (a high confidence score of 0.705 or 70.5%, including the scores from text mining) (Figure 1; Supplementary Table S6).

Furthermore, protein and metabolite fingerprints form protein–metabolite interactions at high confidence such as FBP (FOLR1)–folate (a high confidence score of 0.917 or 91.7%, including the scores from experiments, databases, and text mining), APOA1–linolenic acid (a high confidence score of 0.913 or 91.3%, including the scores from databases and text mining), GOT1–tyrosine (a high confidence score of 0.911 or 91.1%, including the scores from databases and text mining), PLIN2–linolenic acid (a high confidence score of 0.908 or 90.8%, including the scores from databases and text mining), C3–prostaglandin (prostaglandin E2 or PGE2) (a high confidence score of 0.907 or 90.7%, including the scores from databases and text mining), APOA2–linolenic acid (a high confidence score of 0.90 or 90%, including the scores from databases), C3–prostaglandin (prostaglandin D2 or PGD2) (a high confidence score of 0.90 or 90%, including the scores from databases), IGFBP2–prostaglandin (PGF2α) (a high confidence score of 0.804 or 80.4%, including the scores from text mining) (Figure 1; Supplementary Table S6).

### 3.3 MicroRNA–protein interactions in the early pregnancy stages of dairy cows

**FIGURE 2:**
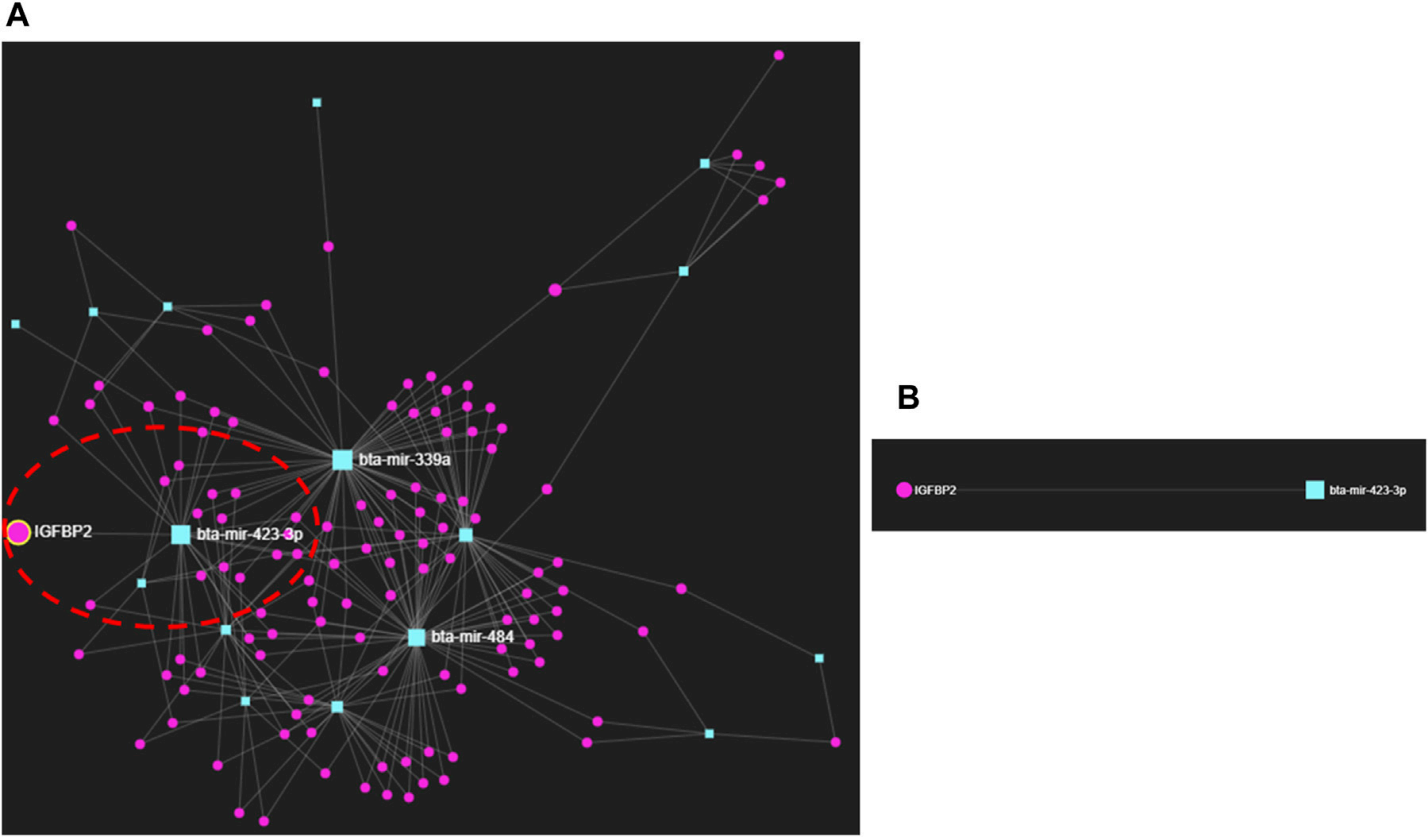
**(A)** miRNA–protein interactions of early pregnancy stages in dairy cows. **(B)** Interaction between bta-miR-423-3p and IGFBP2.

We selected the protein fingerprints that formed protein–metabolite interactomes at high confidence (Figure 1; Supplementary Table S6) and the miRNA fingerprints listed in Table 1 to analyze miRNA–protein interaction networks using the miRNet web tool (http://www.mirnet.ca/) (Fan et al., 2016; Fan and Xia, 2018), with the well-annotated miRanda database (Betel et al., 2010) and proven prediction ability (Enright et al., 2003; Fan et al., 2016). Interestingly, we found an interaction between bta-miR-423-3p and IGFBP2 (Figure 2; Supplementary Table S7). bta-miR-423-3p, the miRNA fingerprint, and IGFBP2, the protein fingerprint, were involved as the early pregnancy fingerprints of dairy cows. The bta-miR-423-3p and IGFBP2 interaction network using the miRNet web tool (http://www.mirnet.ca/) (Fan et al., 2016; Fan and Xia, 2018) was found to have predicted a high miRanda score of 169 and an MFE score of −25.14 (Supplementary Table S7). The MFE score is the minimum free energy score expressed as kcal/mol that explains the binding affinity between miRNAs and their target genes (Rath et al., 2016). An increase in the binding affinity of miRNA and its target genes results in low free energy (Mathews et al., 1999; Kalaigar et al., 2022). An MFE score of −25.14 (Peterson et al., 2014; Rath et al., 2016) explains the strong, stable, and energetically favorable binding affinity between bta-miR-423-3p and IGFBP2.

Furthermore, the interaction between bta-miR-423-3p and IGFBP2 obtained by using miRNet, a web-based platform tool (http://www.mirnet.ca/) (Fan et al., 2016; Fan and Xia, 2018), was also confirmed using the miRWalk database (http://mirwalk.umm.uni-heidelberg.de) (Ding et al., 2016; Sticht et al., 2018; Veshkini et al., 2022), with the site accessibility of 4.42E-05; binding site: 436,457; binding probability (p) of 1 and energy of −25.5; number of pairings: 18; binding region length: 21; longest consecutive pairings: 11; position: CDS; and an ME value of −10.9177 (Supplementary Table S8; Supplementary Figure S1). ME stands for motif m/e, explaining the probabilities of pairing at different miRNA positions.

## 4 Discussion

### 4.1 miRNA–protein–metabolite interaction network in the early pregnancy stages of dairy cows

Interestingly, we saw that the miRNA–protein interaction occurred between bta-miR-423-3p and IGFBP2 with the binding probability (p) of 1 and energy of −25.5, along with the site accessibility of 4.42E-05; binding site: 436,457; number of pairings: 18; binding region length: 21; longest consecutive pairings: 11; position: CDS; and an ME value of −10.9177 found from the miRWalk (http://mirwalk.umm.uni-heidelberg.de/) (Ding et al., 2016; Sticht et al., 2018; Veshkini et al., 2022) database along with the minimum free energy (MFE) score of −25.14 and the predicted high miRanda score of 169 using the miRNet web tool (http://www.mirnet.ca/) (Fan et al., 2016; Fan and Xia, 2018). (Figure 2; Supplementary Tables S7; S8; Supplementary Figure S1).

The miRWalk database (http://mirwalk.umm.uni-heidelberg.de) (Ding et al., 2016; Sticht et al., 2018; Veshkini et al., 2022) generates predicted and validated miRNA-binding sites of known gene interactions. The prediction is made with the random forest-based approach software program TarPmiR (Ding et al., 2016) that searches complete transcript sequences such as 5′-UTR, CDS, and 3′-UTR, along with integration with other well-known databases, including DIANA-microT-CDS, miRanda-rel2010, DIANA-microTv-4.0, miRmap, mirBridge, doRiNA, miRNAMap, PicTar, targetScan, miRDB, miRTarBase, and miRBase (Sticht et al., 2018; Kalaigar et al., 2022).

The miRanda algorithm has similarity to the Smith–Waterman algorithm (Smith and Waterman, 1981). miRanda uses an algorithm that finds complementarity matches between miRNAs and their targets by involving dynamic programming alignment, thermodynamic measurements to evaluate the energetics of physical interactions, and evolutionary conservation as the informational filter (Enright et al., 2003; Peterson et al., 2014). Interestingly, miRanda matches the entire miRNA sequence for miRNA-target prediction (Enright et al., 2003), and mirSVR, which is a support vector regression, gives the scoring that indicates the strength of miRNA’s regulatory effect (Betel et al., 2010; Peterson et al., 2014). In addition, some studies (Enright et al., 2003) have shown that miRanda predictions have also been validated.

Notably, the protein IGFBP2 has also been found to interact with prostaglandin (PGF2α), a metabolite with a high confidence score of 0.804 or 80.4%, including the scores from text mining using the STITCH (Szklarczyk et al., 2016) database (Figure 1; Supplementary Table S6).

**FIGURE 3:**
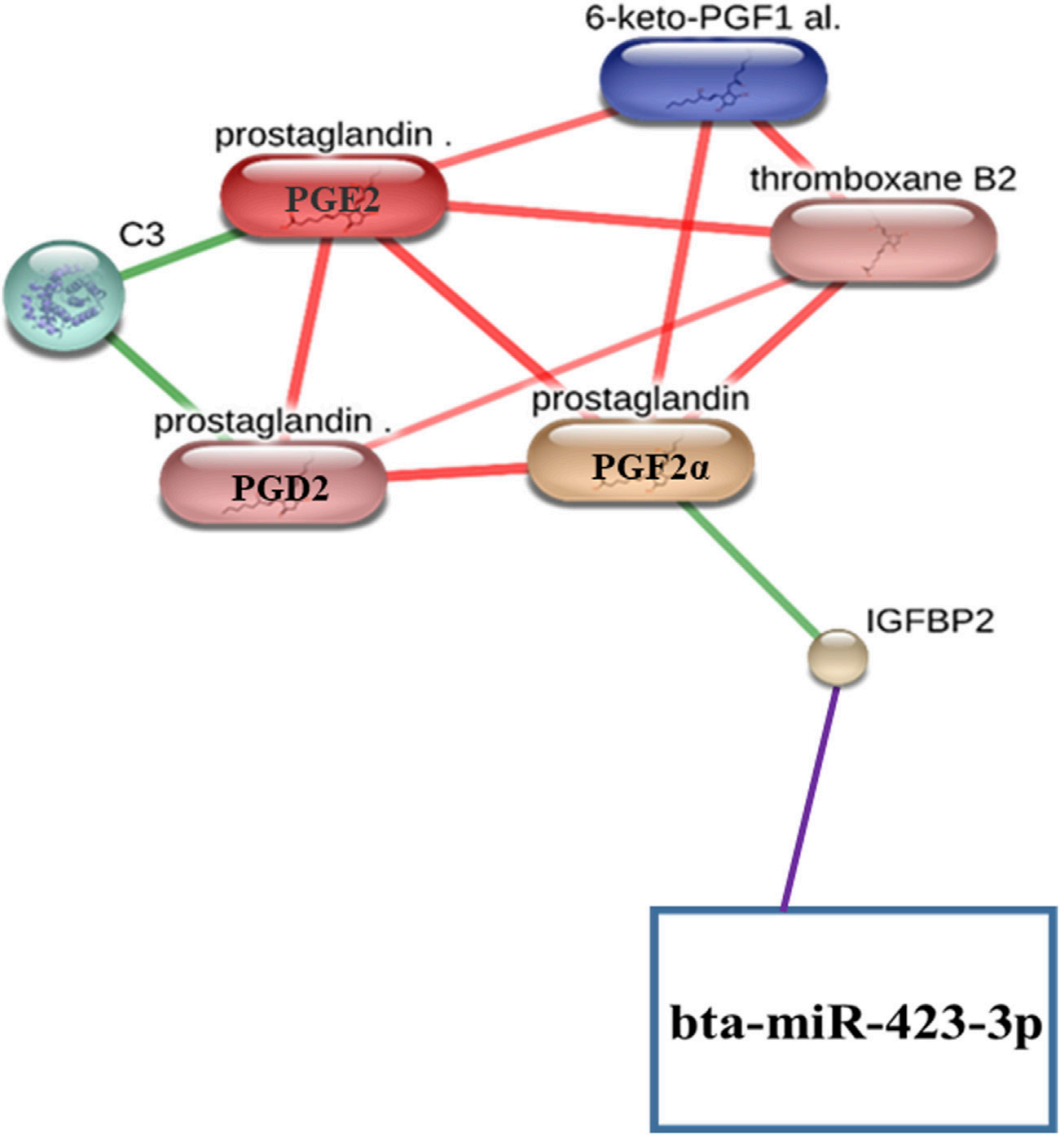
Representation of miRNA–protein–metabolite interactomes. The miRNA–protein interaction is represented in purple, protein–metabolite interactions are represented in green, and metabolite–metabolite interactions are represented in red.

As we found that the protein IGFBP2 interacts with both bta-miR-423-3p (Figure 2; Supplementary Figure S1; Supplementary Tables S7, S8) and prostaglandin (PGF2α) (Figure 1; Supplementary Table S6), we manually integrated the miRNA–protein interaction, such as bta-miR-423-3p and IGFBP2 and protein–metabolite interactions such as IGFBP2 and PGF2α, through the protein IGFBP2, resulting in the formation of miRNA–protein–metabolite interactomes such as bta-miR-423-3p–IGFBP2–PGF2α interactomes (Figure 3). Furthermore, we also found that PGF2α is also interacting with different metabolites, thus forming metabolite–metabolite interactions such as PGF2α–PGD2, PGF2α–thromboxane B2, PGF2α–PGE2, and PGF2α–6-keto-PGF1α at high confidence scores (≥0.7 or 70%). In addition, the interactions between C3–PGE2, C3–PGD2, PGE2–PGD2, PGD2–thromboxane B2, PGE2–thromboxane B2, 6-keto-PGF1α–thromboxane B2, and PGE2–6-keto-PGF1α were also found at high confidence scores (≥0.7 or 70%) (Figure 1; Figure 3; Supplementary Table S6).

## 5 Conclusion

We selected different miRNAs, proteins, and metabolites from the literature that had played an important role in the early stage of pregnancy in dairy cows. Furthermore, we also selected the dairy cows, who were fed the standard diet. We did not consider the pregnancy-related miRNA, protein, and metabolite biomarkers in dairy cows after the transfer of embryos produced by *in vitro* fertilization.

The important pathways, biological processes, molecular functions, and cellular components related to the early pregnancy of dairy cows were enriched by *in silico*-generated target genes for the differentially expressed miRNA fingerprints of the early pregnancy stage in dairy cows.

We manually generated the bta-miR-423-3p–IGFBP2–PGF2α interaction network by manually combining the interaction network formed between bta-miR-423-3p–IGFBP2 and the interaction network between IGFBP2–PGF2α with IGFBP2 as a common interactor with bta-miR-423-3p and PGF2α. Notably, the bta-miR-423-3p–IGFBP2 interaction is found to have many sources of evidence, including a high miRanda score of 169, a minimum free energy (MFE) score of −25.14, binding probability (p) of 1, and energy of −25.5. In addition, the IGFBP2–PGF2α interaction also occurs with high confidence scores (≥0.7 or 70%).

Interestingly, PGF2α is also found to interact with different metabolites, such as PGF2α–PGD2, PGF2α–thromboxane B2, PGF2α–PGE2, and PGF2α–6-keto-PGF1α at high confidence scores (≥0.7 or 70%). Additionally, the interactions between C3–PGE2, C3–PGD2, PGE2–PGD2, PGD2–thromboxane B2, PGE2–thromboxane B2, 6-keto-PGF1α–thromboxane B2, and PGE2–6-keto-PGF1α were also observed at high confidence scores (≥0.7 or 70%).

Therefore, we propose that miRNA–protein–metabolite interactomes involving miRNA, proteins, and metabolites including bta-miR-423-3p, IGFBP2, PGF2α, PGD2, C3, PGE2, 6-keto-PGF1 alpha, and thromboxane B2 found in the early pregnancy stages of dairy cows may form the key regulatory networks and players of pregnancy regulation in dairy cows. These miRNA–protein–metabolite interactomes represent a promising approach for *in silico* biomarker discovery in dairy cow pregnancy and may serve as an alternative to the traditional methods of detection of dairy cow pregnancy.

To the best of our knowledge, this is the first study involving miRNA–protein–metabolite interactomes in the early pregnancy stage of dairy cows. In future, the experimental (*in vivo and in vitro*) studies will be carried out to investigate the bta-miR-423-3p–IGFBP2–PGF2α interactions. In addition, web-based platforms would be developed to integrate miRNA, proteins, and metabolites of organisms/animals together to provide miRNA–protein–metabolite interaction networks.

## Supplementary material

Supplementary Table S1: Summary of the target genes of miRNA fingerprints in dairy cows.

Supplementary Table S2: GO-Slim Biological Process of the target genes of the miRNA fingerprints in dairy cows.

Supplementary Table S3: GO-Slim Molecular Function of the target genes of the miRNA fingerprints in dairy cows.

Supplementary Table S4: GO-Slim Cellular Components of the target genes of the miRNA fingerprints in dairy cows.

Supplementary Table S5: Kegg pathways of the target genes of the miRNA fingerprints in dairy cows.

Supplementary Table S6: Protein-protein, protein-metabolite and metabolite-metabolite interactions between proteins and metabolites fingerprints of early pregnancy stage in dairy cows.

Supplementary Table S7: miRNA – protein interactions in the early pregnancy stage of dairy cows.

Supplementary Table S8: An interaction between bta-miR-423-3p and IGFBP2 found by using miRWalk database in the early pregnancy stage of dairy cows.

Supplementary Figure S1: Details of Duplex from the interaction of bta-miR-423-3p and IGFBP2 in dairy cows obtained from miRWalk database.

## Abbreviations

miRNA, microRNA; ISG15, interferon-stimulated gene-15 ubiquitin-like modifier; AI, artificial insemination.
