# Supplementary figures and images for: miRNA–protein–metabolite interaction network reveals the regulatory network and players of pregnancy regulation in dairy cows
Source: Front Cell Dev Biol. 2024 Aug 2;12:1377172. doi: 10.3389/fcell.2024.1377172 (PMC11329941; doi:10.3389/fcell.2024.1377172)

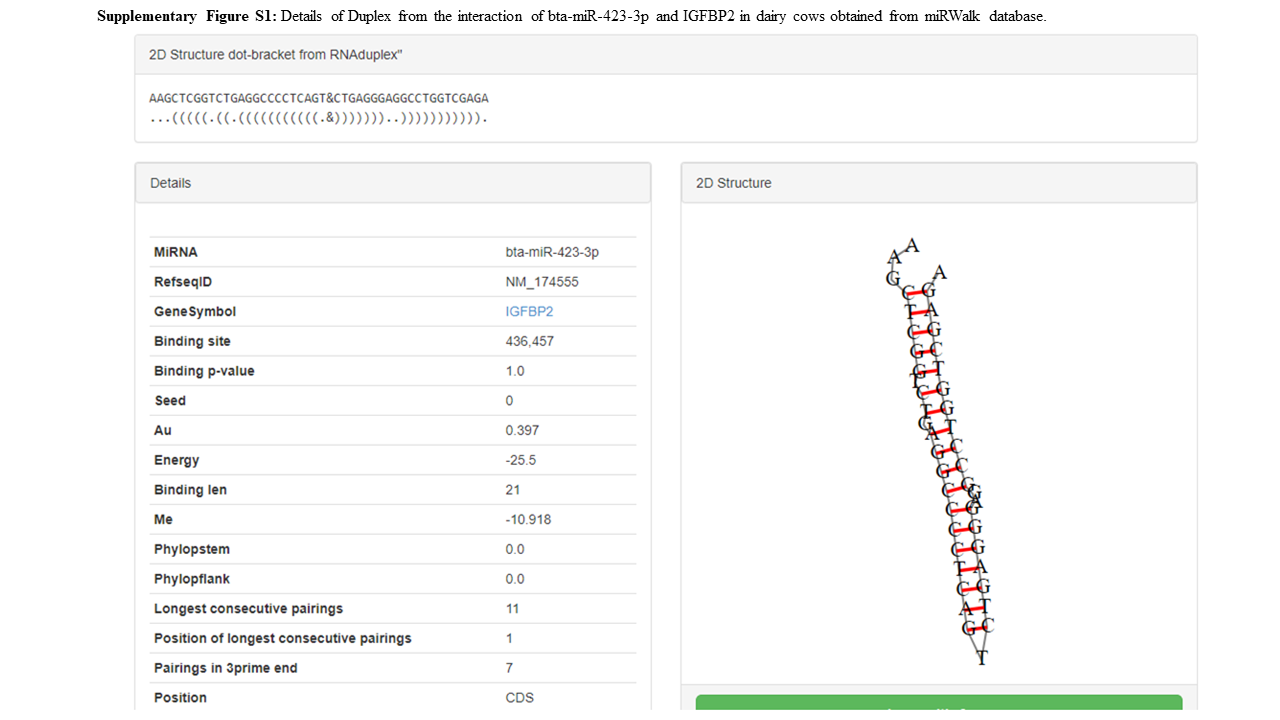

Supplement: Supplementary file 3 [file Image1.tif]
